# Identification, characterization and expression analysis of transient receptor potential channel genes in the oriental fruit fly, *Bactrocera dorsalis*

**DOI:** 10.1186/s12864-018-5053-7

**Published:** 2018-09-14

**Authors:** Hong-ai Su, Xue Bai, Tian Zeng, Yong-yue Lu, Yi-xiang Qi

**Affiliations:** Department of Entomology, College of Agriculture, South China Agricultural University, Wushan Road 483, Tianhe District, Guangzhou, 510642 China

**Keywords:** *Bactrocera dorsalis*, Transient receptor potential, Cationic channels, Splice forms, Expression analysis

## Abstract

**Background:**

Members of the transient receptor potential (TRP) superfamily are proteins that are critical for insects to detect changes in environmental stimuli and also play key roles in their sensory physiology. Moreover, this family provides potential targets for the design of insecticides. In contrast to a large number of studies conducted on *Drosophila melanogaster*, molecular studies to characterize TRP channels in agricultural pests are lacking.

**Results:**

In this study, we identified 15 TRP channel genes in the genome of a notorious agricultural pest, the oriental fruit fly (*Bactrocera dorsalis*). Comparative analysis of the TRP channels (TRPs) in *B. dorsalis* with those in *D. melanogaster*, *Glossina morsitans*, *Musca domestica* and the closely related *Ceratitis capitata*, and TRPs from mosquitoes, Hymenoptera, Lepidoptera, Coleoptera and Hemiptera reveals that members of TRPA and TRPP subfamily are most diverse among insects. The results also suggest that Tephritidae family have two TRP-Polycystin 2 members even though most insects either possess just one or none. The highest expression levels of these two genes are in the testes of *B. dorsalis*, implying a role in regulating sperm function. We analyzed the expression profiles of the TRP channels identified in this study at different life stages using quantitative real time PCR. The results of this study demonstrate that all TRP channels are mainly expressed in adults, especially at mature stages. The one exception to this trend is *BdTRPM*, which is more highly expressed in the eggs of *B. dorsalis*, implying an important role in early development. We also detected the spatial expression of TRP channels in mature adult fruit flies by investigating expression levels within various tissues including those involved in sensory function, such as antennae, compound eyes, mouthparts, legs, and wings, as well as tissues critical for homeostasis and physiology (i.e., Malpighian tubules, the brain and gut as well as fat bodies, ovaries, and testes).

**Conclusion:**

The results of this study establish a solid foundation for future functional characterization of *B. dorsalis* TRP channels as well as those of other insects and will help future insecticide design targeting these channels.

**Electronic supplementary material:**

The online version of this article (10.1186/s12864-018-5053-7) contains supplementary material, which is available to authorized users.

## Background

Transient receptor potential (TRP) superfamily proteins are six transmembrane domain cationic channels with some calcium permeability, implicated in many cellular functions [1]. This superfamily, whose members are found in all animals, can be activated by a variety of mechanisms and play critical roles in sensory physiology including vision, hearing, taste, touch, gravity, olfaction, humidity, thermo- and osmosensation [1, 2]. In insects, these channels have a profound impact on behaviors and physiological functions [2–5].

The first TRP channel to be identified was *Drosophila TRP*; this family member was initially identified and characterized in phototransduction about three decades ago [6]. Numerous TRP-like channels have subsequently been identified in eukaryotes; these are arranged into seven subfamilies based on their primary amino acid sequence homology, TRP-Canonical (TRPC), TRP-Ankyrin (TRPA), TRP-No mechanoreceptor potential C (TRPN), TRP-Vanilloid (TRPV), TRP-Melastatin (TRPM), TRP-Mucolipin (TRPML), and TRP-Polycystin (TRPP) [1, 2]. These seven subfamilies are themselves broadly divided into two groups with TRPC, TRPA, TRPN, TRPV, and TRPM classified as group 1 TRPs because they share the most sequence similarity with the founding member of this superfamily, *Drosophila* TRP. In contrast, group 2 TRPs comprise the TRPP and TRPML subfamilies that are distantly related to their group 1 counterparts [1]. Sixteen TRP members have been identified and characterized in *Drosophila*. The TRPC subfamily comprises TRP, TRP-Like (TRPL), and TRPgamma (TRPγ). The TRPA subfamily includes TRPA1, Painless (Pain), Pyrexia (Pyx), and Waterwitch (wtrw), while the TRPN subfamily has just a single member that is not found in mammals, No mechano-receptor potential C (NompC). The TRPV subfamily includes inactive (Iav) and Nanchung (Nan), while TRPM is the only member of the subfamily bearing this name. The TRPP subfamily has polycystin-2 (Pkd2) and brivido (Brv). In contrast to most TRP members which have 6 transmembrane domains, Brv proteins contain between 8 and 10 transmembrane segments and are not known to form ion channels independently previously [7]. But recently, the *Drosophlia* Brv1 is proved to forms a mechanosensitive cation channel and is essential for gentle-touch sensation [8]. Finally, TRPML subfamilies only contain TRP Mucolipin [1, 2]. Previous research has demonstrated the presence of a diverse range of TRP superfamily members amongst insect species [9]; if the Brv genes are not counted, most insects possess between 13 and 14 TRP components, approximately half the number in mammals [10].

The oriental fruit fly, *Bactrocera dorsalis* (Hendel) (Diptera: Tephritidae), is a polyphagous pest. This species is recognized as one of the most destructive fruit industry pests because of its wide distribution, rapid invasiveness, and the high level of damage it causes [11, 12]. As the long-term and excessive use of chemical insecticides to mitigate the problems caused by this species have led to serious resistance issues [13, 14], it is now urgent to develop alternative targets. In this context, TRP channels have become key insecticide targets because of their critical physiological and cellular functions. Previous researches have shown that both pymetrozine and afidopyropen act by modulating TRPV channels [15, 16], and that TRPA1 in the mosquito vector for malaria, *Anopheles gambiae*, is potently and directly activated by citronellal [17]. Thus, TRPs have become potential targets for insecticide development as well as for improved repellents to control insect-borne diseases [18, 19]. Investigating the molecular characteristics of TRPs in *B. dorsalis* will enable a better understanding of this system in a key agricultural pest and will provide a firm foundation for future insecticide design targeting these channels.

In this study, we identified 15 TRP members in *B. dorsalis* genome and transcriptome data deposited in the GenBank database and examined the expression patterns of these TRPs at different developmental stages and in various *B. dorsalis* tissues.

## Results

### Identification, sequence analysis, and splice variants of TRP channels in *B. dorsalis*

**Fig. 1:**
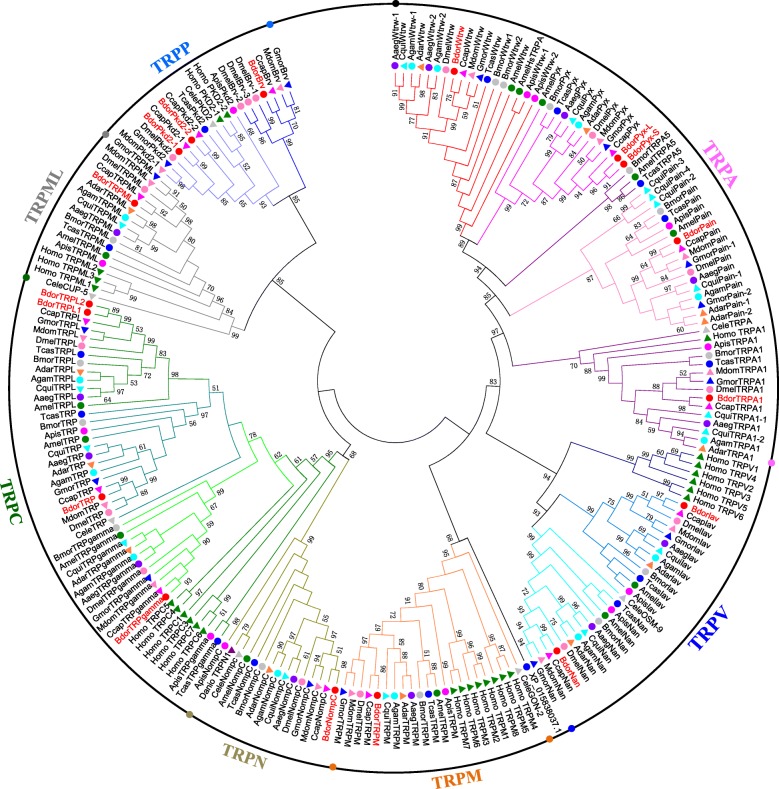
Phylogenetic analysis of TRP channels in *B. dorsalis* and other insects. The tree was constructed using the software MEGA 5.05 with 1000 bootstrap replicates based on the Maximum Likelihood method. The numbers on branch nodes denote levels of bootstrap support. Species abbreviations are Aaeg, *Aedes aegypti*, Adar, *Anopheles darlingi*, Agam, *Anopheles gambiae*, Amel, *Apis mellifera*, Apis, *Acyrthosiphon pisum*, Bdor, *Bactrocera dorsalis*, Bmor, *Bombyx mori*, Ccap, *Ceratitis capitata*, Cqui, *Culex quinquefasciatus*, Cele, *Caenorhabditis elegans*, Dmel, *Drosophila melanogaster*, Danio, *Danio rerio*, Gmor, *Glossina morsitans*, Homo, *Homo sapiens*, Mdom, *Musca domestica*, Tcas, *Tribolium castaneum*

**Fig. 2:**
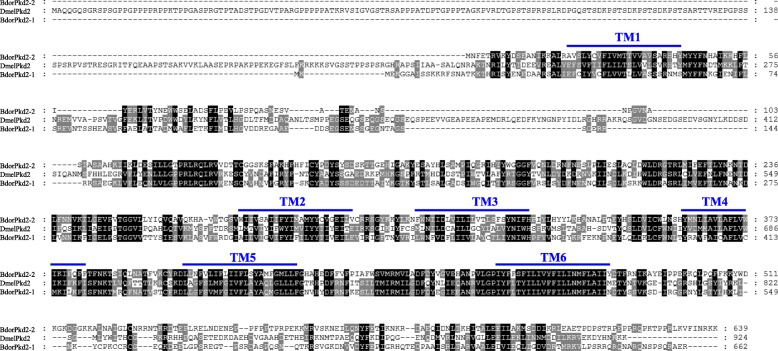
The amino acid sequence alignment of Pkd2 in *B. dorsalis* and *D. melanogaster*. The last residue in each line is indicated on the right, aligned sequences are shown as white letters on black, and conservatively substituted residues are shaded. Dashes indicate gaps introduced to maximize similarities, and the six predicted transmembrane regions are denoted TM1 to TM6

We identified 15 TRP channel genes in *B. dorsalis* that share homology with known *Drosophila* TRP channel sequences. Phylogenetic analysis reveals that these channels include three TRPC, four TRPA, one TRPN, two TRPV, one TRPM, three TRPP, and one TRPML subfamily members, respectively (Fig. 1 and Table 1). Sequence analysis revealed the presence of six transmembrane domains in all *B. dorsalis* TRPs with the exception of BdorBrv, which has eight transmembrane segments (Table 1). Most of group-1 TRPs possess multiple N-terminal ankyrin repeats domain (Table 1); BLASTP analyses of protein sequence alignments show that all *B. dorsalis* TRPs have a high level of sequence identity (above 50%) versus those in *D. melanogaster*, with the exception of the TRPP subfamily members (Table 1). Data show that while *Drosophila* has just one *Pkd2* gene, *B. dorsalis* has two; *BdorPkd2–1* and *BdorPkd2–2*. The Mediterranean fruit fly, *Ceratitis capitata* also possess two *Pkd2* genes, while other insects we investigated just have one or none (Table 2). We validated the ORF sequence of *BdorPkd2–1* and *BdorPkd2–2* via RT-PCR; compared with DmelPkd2, both these genes have a much shorter N-terminal (Fig. 2) and contain a large loop that separates the first two transmembrane domains (Fig. 2), a characteristic feature of group-2 TRPs [1]. No ankyrin repeats were detected in either BdorPkd2–1 or BdorPkd2–2 (Fig. 2 and Table 1). We only identified one *Brv* gene in *B. dorsalis* and other Diptera insects we investigated, with the exception of *D. melanogaster*, which has three (Table 2). Although we also identified one *Brv* gene in *Tribolium castaneum* (XP_015838037.1), it doesn’t cluster with other Brv proteins (Fig. 1). The reason May be that the sequence of the transmembrane segments of this protein is incomplete. To confirm whether Brv proteins are specific to fly species, the genomes of more insect species need to be investigated and the complete sequence of the identified *Brv* gene in *T. castaneum* need to be obtained and characterrized. The numbers of TRP superfamily members among different insect species are varied, even in the same order, such as Diptera (Table 2).

**Table 1:**
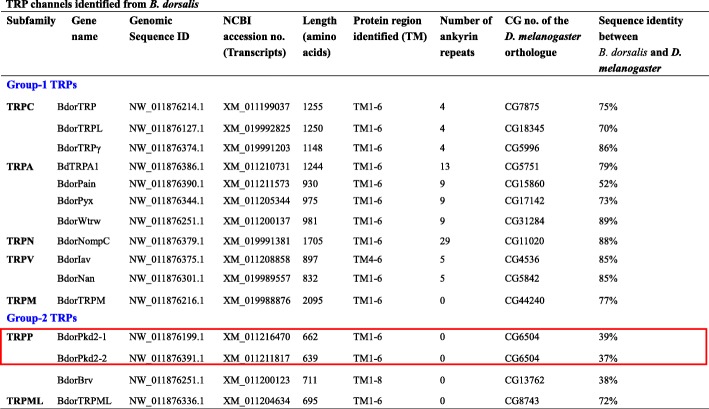
TRP channels identified from *B. dorsalis*

**Table 2:**
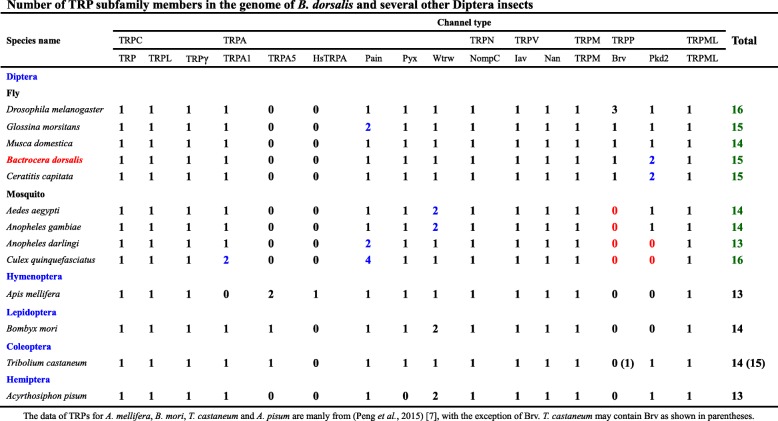
Number of TRP subfamily members in the genome of *B. dorsalis* and several other Diptera insects

The data of TRPs for *A. mellifera*, *B. mori*, *T. castaneum* and *A. pisum* are manly from (Peng et al., 2015) [9], with the exception of Brv. *T. castaneum* may contain Brv as shown in parentheses

**Fig. 3:**
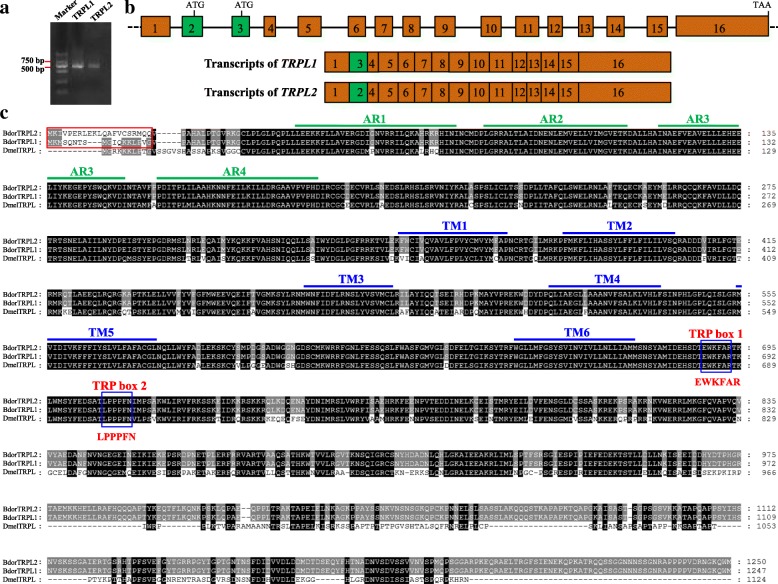
Sequence analysis of the two isoforms of BdTRPL. **a** The two molecular isoforms of *BdTRPL* mRNA detected using PCR. **b** Graphical overview of the *BdTRPL* genomic locus. The rectangles on this figure denote exons, while the lines denote introns. The second exon of *BdTRPL1* and *BdTRPL2* are different from one another, as indicated by green boxes. **c** Comparison of amino acid sequences deduced for BdTRPL1, BdTRPL2, and DmTRPL (*D. melanogaster* TRP). The labels in this figure are the same as in Fig. 2. Ankyrins are denoted AR1 to AR4. TRP box1 and TRP box2 are marked with blue rectangles

As we identified splice variants for *BdorTRPL*, we performed RT-PCR on compound eyes using specific primers and sequenced the products to confirm their form. The results of this step show that at least two splice forms of *BdorTRPL* are present; transcripts of *BdorTRPL1* are more abundant than those of *BdorTRPL2* in the compound eyes of *B. dorsalis* (Fig. 3a). We therefore further analyzed the genome and transcriptome data deposited in the NCBI database to investigate the positions of splicing sites that would be expected to explain the generation of identified forms. The splice forms were generated by two mutually exclusive exons; the second and third (Fig. 3b); thus, the ORF of *BdorTRPL1* is 3744 base pairs (bp) and codes for a protein that comprises 1247 amino-acid residues. Data show that *BdorTRPL2* contains an ORF comprising 3755 bp that codes for a protein consisting of 1250 amino-acid residues. Sequence differences between BdorTRPL1 and BdorTRPL2 include between 18 and 21 N-terminal amino-acid residues (Fig. 3c). The amino acid sequence of BdorTRPL comprises six transmembrane domains (i.e., TM1 to TM6) and four ankyrin repeats. The TRP domain, which follows the sixth transmembrane segment, was detected in BdorTRPL. TRP box 1 and TRP box 2 are the most conserved portions of this domain (Fig. 3c) [1].

**Fig. 4:**
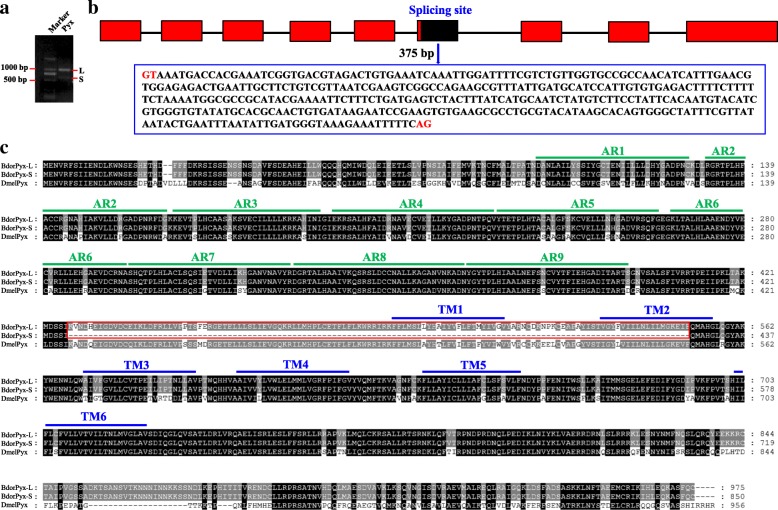
Sequence analysis of the two BdPyx splice forms. **a** Two molecular isoforms of *BdPyx* mRNAs detected via PCR. **b** Cartoon illustration of the two transcripts from *BdPyx*. The shorter of these transcripts includes a truncated exon 6 that originates from the use of an alternative spliced donor site, marked with a black box alongside nucleotide sequences. **c** Comparison of amino acid sequences deduced for BdPyx-L, BdPyx-S, and DmPyx. The labels in this figure are the same as in Fig. 2. Ankyrins are denoted AR1 to AR9. The red box denotes amino acid residues originating from alternative splicing of the *BdPyx* gene

We also detected two splice variants for *BdorPyx* by performing RT-PCR on abdomen samples. Data reveal a higher long form (*BdorPyx-L*) expression level compared to the short form (*BdorPyx-S*) (Fig. 4a); the ORF of *BdorPyx-L* comprises 2928 bp and codes for a protein consisting of 975 amino-acid residues, while that of *BdorPyx-S* is 2553 bp in length and codes for a protein of 850 amino-acid residues. This splicing is generated by the excision of a 375 bp fragment within the sixth exon (Fig. 4b). Thus, compared to the six transmembrane domains seen in BdorPyx-L, this excision is potentially responsible for the generation of the truncated polypeptide in BdorPyx-S that just possesses four (Fig. 4c).

### TRP channel transcript levels in different *B. dorsalis* developmental stages

**Fig. 5:**
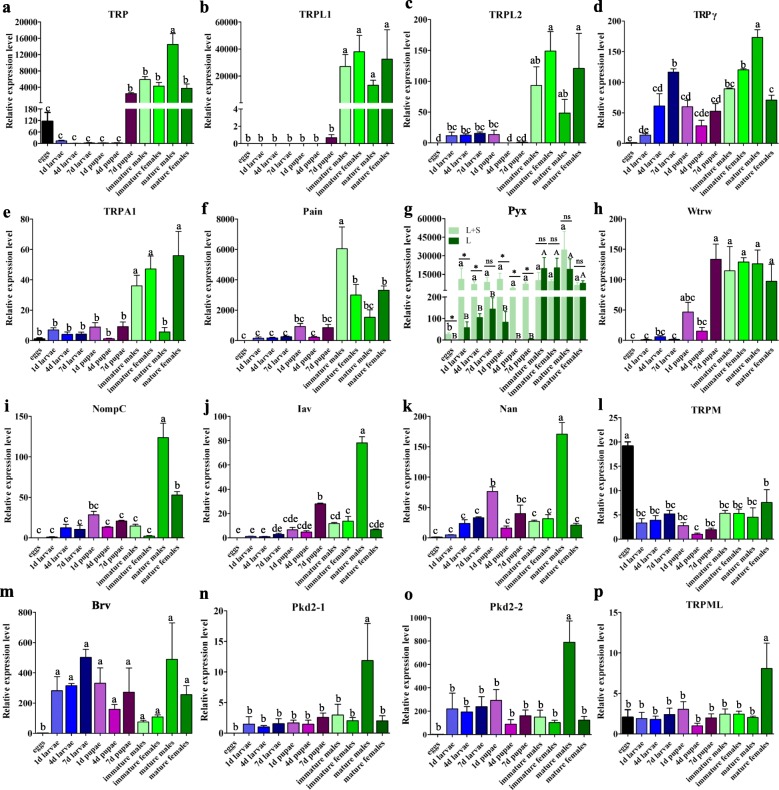
Relative expression level of TRP channels in different developmental stages of *B. dorsalis*, including eggs (one and two hours old), larvae (one day, four days, and seven days old), pupae (one day, four days, and seven days old), one day old immature male and female adults, and 13 day old mature male and female adults. The data presented here are from two independent experiments performed in triplicate. The standard error is represented by the error bar and different letters above each bar denote significant differences (*p* < 0.05) according to ANOVA followed by Tukey’s multiple comparison test. Asterisks indicate values significantly different between the two splice forms of * Pyx* using unpaired t test (*p* < 0.05)

We used qRT-PCR to investigate the levels of temporal expression in *B. dorsalis* TRPs. Multiple stages were tested in this study including eggs, larvae, pupae, immature (one day old), and mature (13 days old) adults. Results reveal that *BdorTRP* is expressed to a relatively high level in adult stages as well as in seven day old pupae (Fig. 5a). Although adults possess abundant transcripts of both the two *BdorTRPL* splice forms (Figs. 5b and c), *BdorTRPL1* is expressed in adults while *BdorTRPL2* is also expressed in larvae and pupae. We were barely able to detect *BdorTRPγ* in eggs even though this form was commonly present in all other tested stages (Fig. 5d). Immature adults also express abundant *BdorTRPA1*, and transcripts of this channel were more common in mature females than males (Fig. 5e). *BdorPain* is more highly expressed in adults than in other stages (Fig. 5f). Results show that the long and short forms of *BdorPyx* were expressed differently depending on *B. dorsalis* life stage; both larval and pupal stages mainly express the short form transcripts while adults mainly express the long form (Fig. 5g). Indeed, similar to *BdorTRP*, abundant *BdorWtrw* transcripts were also detected in seven day old pupae and adults (Fig. 5h), while *BdorNompC*, *BdorIav*, *BdorNan*, *BdorPkd2–1*, and *BdorPkd2–2* were all more highly expressed in mature males compared to all other stages (Figs. 5i, j, k, n and o). *BdorBrv* is widely expressed across all the tested stages except eggs (Fig. 5m). Data show that just *BdorTRPM* was highly expressed in eggs among tested TRPs (Fig. 5l), while mature females expressed the highest number of *BdorTRPML* transcripts (Fig. 5o). All the TRPs we tested are mainly expressed in adults, with the exception of *BdorTRPM* which is expressed to a greater extent in eggs than in other stages.

### Tissue distribution of TRP channels in *B. dorsalis*

**Fig. 6:**
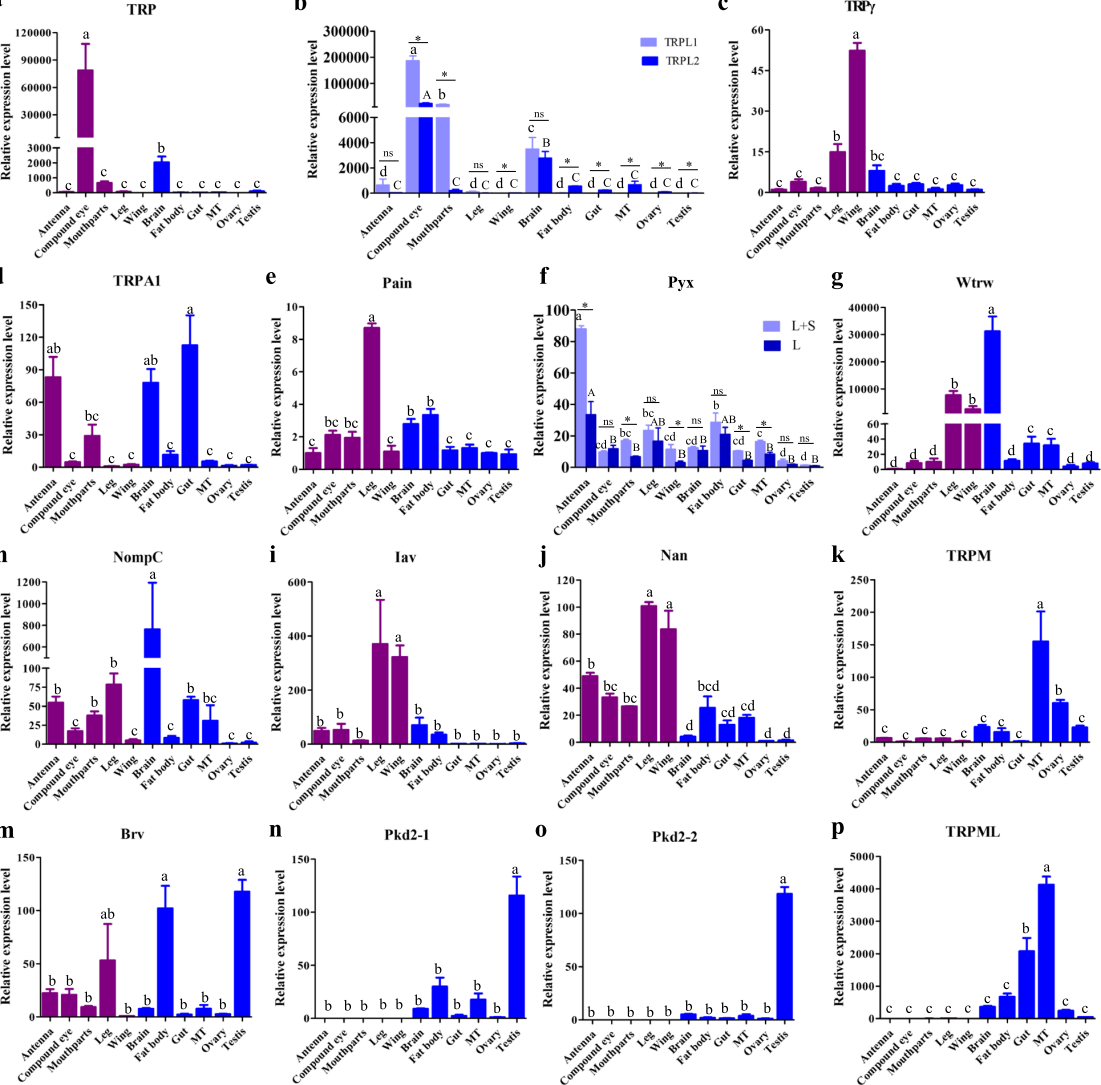
TRP channel relative expression levels in mature adult *B. dorsalis* various tissues. The data presented here are from two independent experiments performed in triplicate. Abbreviation: MT, Malpighian tubules. The standard error is represented by the error bar and different letters above each bar denote significant differences (p < 0.05) according to ANOVA followed by Tukey’s multiple comparison test. Asterisks indicate values significantly different between two splice forms for both *Pyx* and *TRPL* using unpaired t test (*p* < 0.05)

Insect external structures, including antennae, compound eyes, mouthparts, legs, and wings, are all important for sensing environmental stimuli. The internal tissues such as Malpighian tubules, brain, gut and fat bodies as well as ovaries and testes are critical for insect homeostasis. To investigate the possible roles of the identified TRPs in *B. dorsalis*, we therefore measured TRP expression levels in different tissues of mature adults. Results show that transcripts of *BdorTRP*, *BdorTRPL1*, and *BdorTRPL2* are much higher in compound eyes and brains than in other tissues (Figs. 6a-b). In compound eyes and mouthparts, mRNA level of *BdorTRPL1* is higher than that of *BdorTRPL2* (Figs. 6b). While higher transcripts of *BdorTRPL2* were detected in wing, fat body, gut, Malpighian tubules, ovaries and testes than *BdorTRPL1* in these tissues (Figs. 6b). *BdorTRPγ* is mainly expressed in wings, legs and brains (Fig. 6c). In contrast, hardly any transcripts of *BdorTRPA1* were found in wings and legs, but are abundant in antennae, mouthparts, brains and gut (Fig. 6d). Data also show that *BdorPain* is expressed to a high level in legs (Fig. 6e), while *BdorPyx* is mainly expressed in the antennae of *B. dorsalis*. It is also noteworthy that primer-amplified *BdorPyx* comprises two isoforms which coexpressed in this analysis in a ratio strongly favoring the longer variety in compound eyes, legs, brains, fat body, ovaries and testes (Fig. 6f). High messenger RNA (mRNA) levels of *BdorWtrw*, *BdorIav*, and *BdorNan* were also found in legs and wings (Figs. 6g, i and j). The highest expression level of both *BdWtrw* and *BdNompC* were detected in brains (Figs. 6g and h). Abundant transcripts of *BdorTRPM* and *BdorTRPML* were detected in Malpighian tubules (Figs. 6k and p), while a high *BdorTRPM* and *BdorTRPML* mRNA level was also found in ovaries and gut respectively (Fig. 6k and p). The highest expression levels of *BdorBrv*, *BdorPkd2–1* and *BdorPkd2–2* were observed in testes in all cases (Fig. 6m-o). Apart from testes, *BdorBrv* is also highly expressed in fat bodies and legs (Fig. 6m).

## Discussion

We identified 15 *B. dorsalis* TRPs in this study that can be divided into seven subfamilies on the basis of their structure and phylogenetic analyses. Our data show that *B. dorsalis* possesses three TRPP members including BdorBrv, BdorPkd2–1 and BdorPkd2–2. The numbers of TRPP channels are known to vary amongst insect species; while most contain just one *Pkd2* gene (e.g., *Drosophila*), this channel is entirely absent in some lepidopterans and hymenopterans (e.g., *Bombyx* and *Apis*) (Table 2) [10]. *D. melanogaster* have three *Brv* genes, but we detected only one *Brv* gene in *B. dorsalis*. The rest insects we investigated also have one *Brv* or none. It is thought that TRPP comprises the most ancient TRP subfamily because members of this group are known from taxa spanning yeast to mammals [20, 21]. However, the sequence identities of TRPP members between *B. dorsalis* and *D. melanogaster* are relatively low; previous studies have shown that the evolutionary rate of TRPP channels has been accelerated relative to other TRPs and members of this subfamily might perform different physiological functions in distinct insect species [10, 22]. Although *B. dorsalis* have the same number of *TRPA* genes with *D. melanogaster*, members of this subfamily are most diverse among arthropod species, particularly in insects [9]. In this study, more *TRPA* gens were detected in mosquitos than in flies. The *TRPA1* gene was lost in Hymenoptera, but insects belonging to this order have Hymenoptera- specific *TRPA* (*HsTRPA*) probably compensates for the lack of *TRPA1* [10, 23]. TRPA subfamily members are involved in sensing compounds, temperature and humidity. The more complicated lifestyle an insect live, the more TRPA members they may have. Due to the expansion of Pain and TRPA5 channels, the social insect *S. invicta* have 27 TRP genes, much more than most insects [9]. The amplification and reduction of TRPs in different insects indicate that the evolution of this superfamily is related to specific habitats and life histories of individual species. Future studies are needed to investigate the physiological significance of the expanded TRPs.

All of the TRPs detected in this study are highly expressed in adults (especially mature individuals), with the exception of *BdTRPM*. We know that these channels are critical to just about every sensory modality in insects and so impact behaviors as diverse as phototaxis, thermotaxis, gravitaxis, the avoidance of noxious tastants and smells, and proprioception [2]. The biology of *B. dorsalis* adults and the environmental conditions within which they live are more diverse than their other life stages; indeed, multiple behaviors are only seen in adult flies, including courtship, mating, flight, and egg-laying. These observations are important because while most TRPs could barely be detected in eggs, the highest TRPM expression level was found during this life stage; this channel may be required for early *B. dorsalis* development as it is critical for Mg^2+^ and Zn^2+^ homeostasis [24, 25] and members of this subfamily are necessary for initial embryonic development in mice [26].

The data presented here show that *BdTRP* and the two splice forms of *BdTRPL* have high levels of expression in compound eyes and the brain, suggesting their roles in light sensation. *Drosophila TRP* is expressed predominantly within rhabdomeric membranes of photoreceptor cells and is required for light responses [6]. At the same time, TRPL also participates in phototransduction and is responsible for remaining light responses in the *trp* mutant [27]. Interestingly, we detected abundant transcripts of *TRPL1* in the mouthparts, suggesting that this channel might modulate *B. dorsalis* feeding. The two spice forms of *BdorTRPL* are differentially expressed. BdorTRPL2 may regulate the function of fat bodies, gut, Malpighian tubules. The TRPγ channel is also highly enriched in photoreceptor cells in *Drosophila* and is therefore thought to be essential to the phototransduction process [28]. Although no TRPγ expression was observed in previous work in the eyes of *Spodoptera littoralis* [5], we were able to detect low expression in *B. dorsalis* compound eyes. It is also noteworthy that *TRPγ* mRNA is abundant in *B. dorsalis* wings and legs; proprioceptive neurons are distributed in the joints of appendages in fruit flies, including in the legs and wings [29], where TRPγ is expressed in proprioceptive organs and contributes to fine motor control [30]. Moderate *TRPγ* transcripts were detected in the brain of *B. dorsalis*, while in *S. littoralis* and *Periplaneta Americana*, TRPγ is highly expressed in the brain and the central nervous system [5, 31]. TRPA1 is involved in numerous sensory processes, including temperature sensation and the avoidance of noxious heat, aversive odorants, tastants, non-volatile irritants, bright lights, and mechanical stimuli [2, 32]. The presence of sensory organs for smell, hearing, and gravity in insect antennae [2] may therefore explain the relatively high *B. dorsalis TRPA1* expression level in these tissues. Dominant expression of TRPA1 homologs in antennae has been reported previously [33, 34], and we also show robust expression of *TRPA1* in *B. dorsalis* gut samples. TRPA1 channel may play a critical role in *B. dorsalis* gut immune responses because it promotes the expulsion of bacteria from the gut via a uracil/Duox pathway and is also required for intestinal stem cell proliferation in response to oxidative stress [35, 36]. Similar to *TRPA1*, *Pain* is widely distributed in *B. dorsalis* and is also important for the sensation of gravity and the avoidance of noxious heat, mechanical stimulation, and dry environments [2]. We therefore hypothesize an extended role for BdorPain via its involvement in signaling pathways that control various physiological processes. Mechanosensation allows animals to respond to soft touches, noxious sensations, sound, and gravity, and may also contribute to hygrosensation, while Pyx is involved in gravity sensation [2], Wtrw is necessary to detect dry air [37], and NompC is important for light touches, locomotion, and hearing [2, 29]. Organs used for mechanosensation are distributed throughout insect legs and wings, coincident with our findings that relatively high expression levels of *Pyx*, *Wtrw*, and *NompC* also occur in the legs of *B. dorsalis*. Pyx also regulates resistance to noxious heat [38]; the highest level of expression for this channel was detected in *B. dorsalis* antennae. Similarly, the TRPV channels Iav and Nan are expressed in Johnston’s organ and play key roles in gravity and sound sensation [2]; we detected moderate expression levels of both these channels in *B. dorsalis* antennae alongside high levels in legs and wings. The distribution of *Iav* and *Nan* in internal tissues is also indicative of their potential roles in regulating *B. dorsalis* physiological processes; as noted, TRPM is essential for Mg^2+^ and Zn^2+^ homeostasis and *D. melanogaster* knocked out this channel have shortened Malpighian tubules [25]. This gene is also essential for life in these flies as mutations result in the death of pupae [25]. Indeed, *TRPM* is expressed at a high level in *B. dorsalis* Malpighian tubules as well as in several other external structures, suggestive of a role in Mg^2+^ and Zn^2+^ homeostasis and the sensation of environmental stimuli. Studies revealed that *Drosophila* TRPM functions in noxious cold sensation and gentle touch mechanosensation [22]. Given the highly expression level of *Brv* in testes, fat bodies and legs, this gene might regulate *B. dorsalis* sperm function, immune response, taste and mechanosensation. In *Drosophila*, *Brv* genes have been implicated in cool sensation in adults and Brv-1 is required for gentle touch [8, 39]. Previous evidence showed that the antennae contribute to cold sensation [2]. Moderate *Brv* expression level was detected in *B. dorsalis* antennae suggesting this protein may also regulate cool sensation in this species. A single Pkd2 is present in *Drosophila* which localizes to the flagellated sperm tail where it is required for storage [40, 41]. Although TRPP is absent from the genome of several insects such as *Bombyx mori*, *Anopheles darling* and *Culex quinquefasciatus* (Table 2) [9], *B. dorsalis* has two of these channels both expressed to a high level in testes. We hypothesize that these two Pkd2 members perform different roles in sperm function. TRPML participates in locomotion, autophagy, and the clearance of apoptotic cells [2]. This channel is highly expressed in *B. dorsalis* Malpighian tubules and gut, therefore it might regulate the functions of these two tissues in the oriental fruit fly.

## Conclusion

The number and function of TRPs varies amongst insect species [9]. In this study, we have investigated the members of TRPs in the genome of the notorious agricultural pest, *B. dorsalis* and several other Diptera species. We also detected the expression patterns of TRPs in *B. dorsalis*. The results of this research expand our knowledge of these critical sensory channels and provide key additional information that will prove valuable to future molecular-level functional studies. This study also lays the foundations for the future development of novel strategies to safely and efficiently control this key insect pest species.

## Methods

### Insects

Individual *B. dorsalis* flies were reared at 27 °C ± 1 °C and at 75% ± 1% relative humidity; all individuals were subjected to a photoperiod cycle comprising 14 h of light and ten hours of dark. Hatched larvae were maintained on an artificial diet [42]. Larvae were then transferred into small plastic boxes containing sand before pupation and pupae were kept at 27 °C ± 1 °C until adults emerged. Adult flies were fed another artificial diet comprising yeast extract and dry sugar mixed at a 1:1 ratio (*w*/w) and housed in wooden cages measuring 35 cm by 35 cm by 35 cm [43].

### Identification of TRP channels

To search exhaustively all TRP genes in each species, we screened several types of database including assembled genomes, reference sequence (RefSeq) database from National Center for Biotechnology Information (NCBI) (https://www.ncbi.nlm.nih.gov/refseq/) and transcriptomic data acquired from NCBI Sequence Read Archive (SRA) Databases (https://www.ncbi.nlm.nih.gov/genbank/tsa/). The *B. dorsalis* genome has been available from the United States Department of Agriculture National Agricultural Library Database (https://i5k.nal.usda.gov/Bactrocera_dorsalis). The genomes of *Glossina morsitans*, *Aedes aegypti, Anopheles darling, Anopheles gambiae* and *Culex quinquefasciatus* has been available from vectorBase (https://www.vectorbase.org/). The genomes of *Ceratitis capitata* [44] and *Musca domestica* [45] were downloaded from NCBI database. We obtained the genome data of *Apis mellifera*, *Bombyx mori*, *Tribolium castaneum*, *Acyrthosiphon pisum* from Hymenoptera Genome database (http://hymenopteragenome.org/), Silkworm Genome database (http://silkworm.genomics.org.cn/), Beetlebase (http://www.beetlebase.org/) and AphidBase (http://bipaa.genouest.org/is/aphidbase/) respectively. Firstly, candidate *B. dorsalis* TRP genes were identified by TBLASTN searches against genome and transcriptomes with an E-value cutoff of 1e^− 5^, using known TRP protein sequences of *D. melanogaster*, *Apis mellifera* and humans (*Homo sapiens*). Then, candidate genes were further verified using BLASTP versus non-redundant NCBI protein sequences without species limits and with a cut-off e-value of 1e^− 5^ [46]. The same procedure was used to identify TRP genes of other Diptera species and *Brv* genes of *A. mellifera*, *B. mori*, *T. castaneum*, *A. pisum* by a homology-based approach.

### Reverse transcription PCR (RT-PCR)

To investigate the splice variants of TRP channels and to confirm the identified Pkd2 channels in *B. dorsalis*, primers (Additional file 1: Table S1) were designed to amplify part of the open reading frames (ORFs) of *TRPL*, *Pyx * and *Pkd2* genes. Total RNA was isolated from the compound eyes, abdomens, and whole bodies of six adult flies using the Trizol reagent (Invitrogen, Carlsbad, CA, USA) and was treated with RQ1 DNase I (Promega, Madison, WI) to eliminate genomic DNA (gDNA). Single-strand complementary DNA (cDNA), synthesized from the Total RNA (1 μg) using a RevertAid First Strand cDNA Synthesis Kit (Thermo Scientific), was then used as a template for PCRs. All amplifications were carried out using Phusion high-fidelity DNA polymerase (Thermo Scientific) according to the manufacturer’s instructions and products were separated to check their sizes via electrophoresis onto a 1.0% agarose gel. Purified PCR products were then cloned into a pEASY-Blunt Zero Cloning Vector (TransGen, Beijing, China) following the manufacturer’s instructions before being sequenced.

### Phylogenetic analysis and sequence alignment

In order to classify the TRP channels we identified into different subfamilies, amino acid sequences were phylogenetically characterized in each case. Thus, protein structural information for all candidate TRPs was identified via an InterProScan (http://www.ebi.ac.uk/Tools/InterProScan/) search, and sequences were aligned using the software ClustalW2 (http://www.ebi.ac.uk/Tools/msa/clustalw2/). Phylogenetic trees were constructed using MEGA 5.05 with 1000 bootstrap replicates based on the Maximum Likelihood method. Poisson correction model and the partial deletion method for gaps were used. Branch support values were expressed as percentages. The accession numbers of all the TRP channels used in this study are listed in Additional file 1: Table S2.

### Quantitative real time PCR (qRT-PCR)

Samples from different developmental stages were collected to investigate the spatiotemporal distribution of TRPs in *B. dorsalis*; these included eggs (between one hour and two hours old), larvae (one day, four days, and seven days old), pupae (one day, four days, and seven days old), immature males and females (one day old), and mature males and females (13 days old). 13-day- old adults mixed with the same number of males and females were dissected into antennae, compound eyes, mouthparts, legs, wings, the brain, fat bodies, gut, Malpighian tubules, ovaries and testes. For temporal distribution analysis, approxmatily 100 eggs were used for a pool and 6 were used for a pool for the rest stages. For tissue distribution, 30 adults were included in a pool. The replicates are different pools of individuals from independent cages on the same day. At least three sample biological replicates were carried out in each case.

We extracted RNA using the TRIzol reagent (as discussed above) and measured RNA quantities using a Nanodrop 2000 spectrophotometer (Thermo Scientific Inc., Bremen, Germany). Reverse transcription was then performed with 1 μg of RNA using TransScript one-step gDNA removal and cDNA Synthesis SuperMix (TransGen Biotech, China). Synthesized cDNA was then used as a template for qRT-PCR; this was performed with a Stratagene Mx3000P thermal cycler (Agilent Technologies, Wilmington, DE). The reaction mixtures used in each case contained 12.5 μL of 2 × TransStart Top Green qPCR SuperMix (TransGen Biotech, Beijing, China), 0.4 μL of positive reference dye, 0.4 μL of each primer (0.2 μM), and 2 μL of template cDNA. Sterile distilled water was then added to these mixtures up to a final volume of 25 μL. The thermal cycling conditions used in this study comprised 30 s at 95 °C, 40 cycles at 95 °C for five seconds each, and 34 s at 60 °C. Three sample replicates were performed for each group, and no-template negative controls were included in each run to detect possible contamination or carryover. A series of gene-specific primers were designed for qRT-PCR using the software Primer 3 (http://bioinfo.ut.ee/primer3-0.4.0/) (Additional file 1: Table S1); these primers were utilized to investigate the relative expression of selected samples, while a melting curve analysis was performed between 60 °C to 95 °C for all reactions to ensure the specificity and consistency of generated products. The specificity of all qRT-PCR reaction products was established via electrophoresis on a 1.0% agarose gel prior to sequencing, and all experiments were performed independently at least twice to ensure their reliability and reproducibility.

We quantified the transcript levels of different genes using the 2^-ΔΔCT^ method [47], and ensured comparable quantities of cDNA by amplifying α-tubulin as a reference gene as this possesses excellent spatiotemporal expression stability in *B. dorsalis* [48]. We set the lowest expression level stage to one for this analysis in order to calibrate relative levels in different development stages; relative expression levels were therefore assessed by comparing the situation in each target gene in other developmental stages to that of the lowest stage. The same developmental stage method was also applied for analysis of relative expression levels in various tissues. The data of relative expression levels in different development stages and various tissues were analyzed using one-way analysis of variance (ANOVA), followed by a Tukey’s multiple comparison test when significant differences were tested. For the comparison of expression differences between splice forms, unpaired t test were applied. All statistical analyses were performed using the software GraphPad Prism 5.0 (San Diego, CA).

## Additional file

Additional file 1: **Table S1.** Primers used in this study. **Table S2.** Accession number of TRP channels used in this study. (DOCX 28 kb)
