# Supplementary material for: Identification, characterization and expression analysis of transient receptor potential channel genes in the oriental fruit fly, Bactrocera dorsalis
Source: BMC Genomics. 2018 Sep 14;19:674. doi: 10.1186/s12864-018-5053-7 (PMC6137742; doi:10.1186/s12864-018-5053-7)
Supplement: Supplementary file 1 — Table S1. Primers used in this study. Table S2. Accession number of TRP channels used in this study. (DOCX 28 kb) [file 12864_2018_5053_MOESM1_ESM.docx]

**Table S1 Primers used in this study**

| **RT-PCR** | | |
| --- | --- | --- |
| **Primer name** | **Sequence (5’-3’)** | |
| Bdor Pkd2-1-F | GCTCATCTACAACATTTGGCAT | |
| Bdor Pkd2-1-R | GTTGACGCCGACTTGGTAA | |
| Bdor Pkd2-2-F | GCGTTGGAGAAATACTGGTGT | |
| Bdor Pkd2-2-R | TCTCCTCTAAAGCCGTGATGT | |
| BdorPyx-F | GCTGCAATCGTCAAACAATCT | |
| BdorPyx-R | TGGATAGTCGTTGAATAGCACC | |
| BdorTRPL1/2- F | GTCAAGTGCTGGCAATACAAAC | |
| BdorTRPL1-R | CCACTGGCAGCTTCTTCTGTA | |
| BdorTRPL2-R | CATTCTGGAGCAAACAAATGC | |
| **Real-time PCR** | | |
| **Primer name** | **Sequence (5’-3’)** | **Length (bp)** |
| BdorTRP-q-F | GCAAGGGTGATTGGCAAGAA | 226 |
| BdorTRP-q-R | TCACATCACCCAAGTTCGGA |  |
| BdorTRPL1-q-F | CAGAAGAAGCTGCCAGTGG | 212 |
| BdorTRPL1-q-R | CCCAACGGATCCATGCAATT |  |
| BdorTRPL2-q-F | AGAGAAACTGCAGGCATTTGT | 156 |
| BdorTRPL2-q-R | ATATCACCGCGCTCCACC |  |
| BdorTRPγ-q-F | TTGGACAGACAATGCGCAAA | 225 |
| BdorTRPγ-q-R | CGGAAACCCATGCCAGTATG |  |
| BdorTRPA1-q-F | TGGAGCTCTGCCTGAAATCA | 236 |
| BdorTRPA1-q-R | GCCCTCCTTCACCAAGTACT |  |
| BdorPain-q-F | CAGGTGAATTCGATGCTGGG | 207 |
| BdorPain-q-R | TCAGGACTTGTTCGTAGCGT |  |
| BdorPyx-(L+S) -q-F | CTTGAGATCGAGGAGACGCT | 196 |
| BdorPyx-(L+S) -q-R | CCTTACAATTGGGATCGGCG |  |
| BdorPyx-L-q-F | GCGTACATAAGCACAGTGGG | 150 |
| BdorPyx-L- q-R | CAGCAAGACACCAAACACGA |  |
| BdorWtrw-q-F | AGAGGCCCAATATCCGACAC | 161 |
| BdorWtrw-q-R | GATCGATGAGACTGATGCGC |  |
| BdorNompC-q-F | CAAGTACTTCTACGGCGTGC | 224 |
| BdorNompC-q-R | CGCCCTTCTTGAAGAGCATC |  |
| BdorIav-q-F | AGTCCGCTATTGTGCAGAGT | 197 |
| BdorIav-q-R | CTGTATCGCCCATCAAACGG |  |
| BdorNan-q-F | GCGCATTGTACTGGTAGTGG | 167 |
| BdorNan-q-R | TGCGTTTCATCTCTTGCACC |  |
| BdorTRPM-q-F | TGTTGGTGCAGCTGTTTACC | 183 |
| BdorTRPM-q-R | CACCTGTATTTGTACCGCCG |  |
| BdorPkd2-1-q-F | GCCTCTTCTTCACGCAACAT | 242 |
| Bdor Pkd2-1-q-R | CCCTCACCGCTGCTATTTTC |  |
| Bdor Pkd2-2-q-F | AATGAGTGGTGGTCGGAACT | 239 |
| Bdor Pkd2-2-q-R | TTCGCGAAACTCTTGCTACC |  |
| BdorBrv-q-F | TCAACCAGGGACACGACTAC | 248 |
| BdorBrv-q-R | ATCCATTTGTGCCGCTAACC |  |
| BdorTRPML-q-F | AGAGTTACGGTGGAGCAACA | 185 |
| BdorTRPML-q-R | TTCACCACTGCTTTCATGCC |  |
| α-Tubulin-q-F | CGCATTCATGGTTGATAACG | 184 |
| α-Tubulin-q-R | GGGCACCAAGTTAGTCTGGA |  |

**Table S2 Accession number of TRP channels used in this study.**

| **Gene name** | **Accession number** |
| --- | --- |
| AmelTRP | XP_001120503.2 |
| AmelTRPL | XP_003250136.1 |
| AmelTRPgamma | XP_394299.5 |
| AmelTRPA5 | XP_001122445 |
| AmelPain | XP_001122160 |
| AmelPyx | GB45663 |
| AmelWtrw | XP_395234.3 |
| AmelNompC | XP_392309.1 |
| AmelIav | XP_001121881.1 |
| AmelNan | XP_625170.3 |
| AmelTRPM | XP_395849.4 |
| AmelHsTRPA | XP_395235.2 |
| AmelTRPML | XP_624283.3 |
|  |  |
| ApisTRP | XP_003240303.1 |
| ApisTRPL | XP_003241694.1 |
| ApisTRPgamma | XP_001942539.2 |
| ApisTRPA1 | XP_001944501.2 |
| ApisPain | XP_001950177.2 |
| ApisWtrw1 | ACYPI23235-PA |
| ApisWtrw2 | ACYPI004217-PA |
| ApisNompC | XP_001945728.2 |
| ApisIav | XP_001950096.1 |
| ApisNan | XP_001947907.2 |
| ApisTRPM | XP_003245193.1 |
| ApisPkd2 | XP_008180465.2 |
| ApisTRPML | XP_001949417.2 |
|  |  |
| AaegTRP | AAEL005437 (vectorbase) |
| AaegTRPL | AAEL005575 |
| AaegTRPgamma | AAEL005014 |
| AaegTRPA1 | AAEL001268 |
| AaegPain | AAEL006835 |
| AaegPyx | AAEL004179 |
| AaegWtrw-1 | AAEL027478 |
| AaegWtrw-2 | AAEL023040 |
| AaegNompC | AAEL019818 |
| AaegIav | AAEL020482 |
| AaegNan | AAEL001123 |
| AaegTRPM | AAEL023985 |
| AaegTRPML | AAEL001557 |
|  |  |
| AgamTRP | AGAP000348 (vectorbase) |
| AgamTRPL | AGAP010630 |
| AgamTRPgamma | AGAP008435 |
| AgamTRPA1 | AGAP004863 |
| AgamPain | AGAP013463 |
| AgamPyx | AGAP000107 |
| AgamWtrw-1 | AGAP000361 |
| AgamWtrw-2 | AGAP010269 |
| AgamNompC | AGAP008559 |
| AgamIav | AGAP000413 |
| AgamNan | AGAP012241 |
| AgamTRPM | AGAP006825 |
| AgamTRPML | AGAP007710 |
|  |  |
| AdarTRP | ADMH02000837 |
| AdarTRPL | ADAC010362 |
| AdarTRPgamma | ADAC005418 |
| AdarTRPA1 | ADAC002915 |
| AdarPain-1 | ADAC005756 |
| AdarPain-2 | ADAC007113 |
| AdarPyx | ADAC001291 |
| AdarWtrw | ADAC001843 |
| AdarNompC | ADAC010174 |
| AdarIav | ADAC004108 |
| AdarNan | ADAC010507 |
| AdarTRPM | ADAC010606 |
| AdarTRPML | ADAC004907 |
|  |  |
|  |  |
| BdorTRP | XP_011197339.1 |
| BdorTRPL1 | XP_011213562.1 |
| BdorTRPL2 | XP_019848384.1 |
| BdorTRPgamma | XP_011207043.1 |
| BdorTRPA1 | XP_011209033.1 |
| BdorPain | XP_011209875.1 |
| BdorPyx-L | XP_011203646.1 |
| BdorPyx-S | XP_019845914.1 |
| BdorWtrw | XP_011198439.1 |
| BdorNompC | XP_019846940.1 |
| BdorIav | XP_011207160.1 |
| BdorNan | XP_019845116.1 |
| BdorTRPM | XP_019844435.1 |
| BdorPkd2-1 | XP_011214772.1 |
| BdorPkd2-2 | XP_011210119.1 |
| BdorBrv | XP_011198425.1 |
| BdorTRPML | XP_011202936.1 |
|  |  |
| BmorTRP | BGIBMGA009272-PA |
| BmorTRPL | BGIBMGA009273-PA |
| BmorTRPgamma | BGIBMGA001085-PA |
| BmorTRPA1 | BGIBMGA002131-PA |
| BmorPain | BGIBMGA001166-PA |
| BmorPyx | BGIBMGA005170-PA |
| BmorTRPA5 | BGIBMGA014186-PA |
| BmorWtrw1 | BGIBMGA010027-PA |
| BmorWtrw2 | BGIBMGA010059-PA |
| BmorNompC | BGIBMGA005195-PA |
| BmorIav | BGIBMGA003369-PA |
| BmorNan | BGIBMGA004308-PA |
| BmorTRPM | BGIBMGA001988-PA |
| BmorTRPML | BGIBMGA012072-PA |
|  |  |
| CcapTRP | XP_004536899 |
| CcapTRPL | XP_023159188 |
| CcapTRPgamma | XP_012161545 |
| CcapTRPA1 | XP_020717897 |
| CcapPain | XP_004522641 |
| CcapPyx | XP_004533435 |
| CcapWtrw | XP_012158622 |
| CcapNompC | XP_020715699 |
| CcapIav | XP_004529475 |
| CcapNan | XP_004537742 |
| CcapTRPM | XP_020712713 |
| CcapPkd2-1 | XP_004523517 |
| CcapPkd2-2 | XP_012159700 |
| CcapBrv | XP_004529367 |
| CcapTRPML | XP_004518695 |
|  |  |
| CquiTRP | CPIJ018599 (vectorBase) |
| CquiTRPL | CPIJ005741 |
| CquiTRPgamma | CPIJ001523 |
| CquiTRPA1-1 | CPIJ002323 |
| CquiTRPA1-2 | CPIJ002324 |
| CquiPain-1 | CPIJ006747 |
| CquiPain-2 | CPIJ014495 |
| CquiPain-3 | CPIJ002572 |
| CquiPain-4 | CPIJ002574 |
| CquiPyx | CPIJ010619 |
| CquiWtrw | CPIJ009770 |
| CquiNompC | CPIJ000429 |
| CquiIav | CPIJ014201 |
| CquiNan | CPIJ005713 |
| CquiTRPM | CPIJ014513 |
| CquiTRPML | CPIJ011507 |
|  |  |
| DmelTRP | CG7875 (flybase) |
| DmelTRPL | CG18345 |
| DmelTRPgamma | CG5996 |
| DmelTRPA1 | CG5751 |
| DmelPain | CG15860 |
| DmelPyx | CG17142 |
| DmelWtrw | CG31284 |
| DmelNompC | CG11020 |
| DmelIav | CG4536 |
| DmelNan | CG5842 |
| DmelTRPM | CG44240 |
| DmelBrv-1 | CG9472 |
| DmelBrv-2 | CG16793 |
| DmelBrv-3 | CG13762 |
| DmelPkd2 | CG6504 |
| DmelTRPML | CG8743 |
|  |  |
| GmorTRP | GMOY011996 (vectorBase) |
| GmorTRPL | GMOY012064 |
| GmorTRPgamma | GMOY012137 |
| GmorTRPA1 | GMOY007826 |
| GmorPain-1 | GMOY001967 |
| GmorPain-2 | GMOY006392 |
| GmorPyx | GMOY003140 |
| GmorWtrw | GMOY005391 |
| GmorNompC | GMOY003722 |
| GmorIav | GMOY006587 |
| GmorNan | GMOY001279 |
| GmorTRPM | GMOY005151 |
| GmorBrv | contig ctg10001916 |
| GmorPkd2 | GMOY008881 |
| GmorTRPML | GMOY011941 |
|  |  |
|  |  |
| MdomTRP | XP_011292356 |
| MdomTRPL | XP_011295208 |
| MdomTRPgamma | XP_011295823 |
| MdomTRPA1 | XP_005190946 |
| MdomPain | XP_005178197 |
| MdomPyx | XP_005178657 |
| MdomWtrw | XP_005183123 |
| MdomNompC | XP_019891985 |
| MdomIav | XP_005181017 |
| MdomNan | XP_005180489 |
| MdomTRPM | XP_019895333 |
| MdomPkd2 | XP_019892736 |
| MdomBrv | XP_019892575 |
| MdomTRPML | XP_005177219 |
|  |  |
| TcasTRP | XP_968670.2 |
| TcasTRPL | XP_968598.1 |
| TcasTRPgamma | XP_970049.1 |
| TcasTRPA1 | XP_015834257.1 |
| TcasPain | NP_001164308.1 |
| TcasPyx | XP_972539.2 |
| TcasWtrw | XP_966629.2 |
| TcasTRPA5 | XP_008192024.1 |
| TcasNompC | XP_008197616.1 |
| TcasIav | XP_015838708.1 |
| TcasNan | XP_967896.1 |
| TcasTRPM | XP_974857.2 |
| TcasBrv | XP_015838037.1 |
| TcasPkd2 | XP_969891.2 |
| TcasTRPML | XP_966660.1 |
|  |  |
| CeleTRP | NP_001022703 |
| CeleNompc | NP_493429 |
| CeleGON-2 | NP_502118 |
| CeleOSM-9 | NP_501172 |
| CeleTRPA | NP_001294083 |
| CelePKD2 | NP_502838 |
| CeleCUP-5 | NP_001022721 |
|  |  |
| Danio TRPN1 | NP_899192.1 |
|  |  |
| Homo TRPC1 | NP_003295 |
| Homo TRPC2 | NP_001124170 |
| Homo TRPC3 | NP_001124170 |
| Homo TRPC4 | NP_003297 |
| Homo TRPC5 | NP_036603 |
| Homo TRPC6 | NP_004612 |
| Homo TRPC7 | NP_065122 |
| Homo TRPM1 | NP_002411 |
| Homo TRPM2 | NP_002411 |
| Homo TRPM3 | XP_011517347 |
| Homo TRPM4 | NP_060106 |
| Homo TRPM5 | XP_016873117 |
| Homo TRPM6 | XP_011516546 |
| Homo TRPM7 | NP_001288141 |
| Homo TRPM8 | XP_011510112 |
| Homo TRPV1 | NP_542435 |
| Homo TRPV2 | NP_057197 |
| Homo TRPV3 | NP_001245134 |
| Homo TRPV4 | NP_001170902 |
| Homo TRPV5 | NP_062815 |
| Homo TRPV6 | NP_061116 |
| Homo TRPA1 | NP_015628 |
| Homo PKD2-1 | NP_057196 |
| Homo PKD2-2 | NP_055201 |
| Homo TRPML1 | NP_065394 |
| Homo TRPML2 | NP_694991 |
| Homo TRPML3 | NP_001240622 |
